# ﻿Description of two new species of the genus *Trochus* Linnaeus, 1758 (Gastropoda, Trochidae) from the South China Sea

**DOI:** 10.3897/zookeys.1264.167854

**Published:** 2025-12-19

**Authors:** Peng-Jin Zhu, Cheng-Rui Yan, Hong-Qiang Yang, Li-Sha Hu, Yun-Wei Dong

**Affiliations:** 1 Key Laboratory of Mariculture, Ministry of Education, Ocean University of China, Qingdao 266003, China; 2 Shandong Key Laboratory of Green Mariculture and Smart Fishery, Fisheries College, Ocean University of China, Qingdao 266003, China; 3 Key Laboratory of Ocean and Marginal Sea Geology, South China Sea Institute of Oceanology, Chinese Academy of Sciences, Guangzhou 510301, China; 4 Southern Marine Science and Engineering Guangdong Laboratory (Guangzhou), Guangzhou 510301, China; 5 Frontiers Science Center for Deep Ocean Multispheres and Earth System, Qingdao 266003, China

**Keywords:** 3D modeling, geometric morphometrics, mitochondrial COI gene, new species, South China Sea, Top shells, *
Trochus
*

## Abstract

Two new species of the gastropod family Trochidae, *Trochus nanhai***sp. nov.** and *Trochus parvus***sp. nov.**, are described from the South China Sea. Morphological comparisons and species delimitation analyses based on molecular phylogeny support the distinctiveness of the two species. *Trochus nanhai***sp. nov.** exhibits substantial variation in shell morphology, which increases the difficulty of morphological characterization. Nevertheless, it can be distinguished by diagnostic characters such as the presence and arrangement of pustules along the suture of each whorl. Additionally, multiple species delimitation methods (ASAP, ABGD, bPTP and GMYC) revealed potential taxonomic inconsistencies within previously identified *Trochus* species. To assist visualization and facilitate future taxonomic studies, we applied 3D modeling techniques and extracted geometric morphometric parameters of two new species. Detailed morphological descriptions, diagnostic characters, illustrations, and 3D models are provided. This study enhances our understanding of *Trochus* diversity and taxonomy.

## ﻿Introduction

Trochidae Rafinesque, 1815 is a large heterogeneous family of gastropods, with species distributed globally, predominantly in tropical and subtropical regions (Williams et al. 2008). Trochidae, a diverse family within the superfamily Trochoidea, comprises more than 2000 extant species grouped into approximately 500 recognized genera (Hickman et al. 1996; Ponder and Lindberg 2008; Williams et al. 2010). Within Trochidae, the genus *Trochus* Linnaeus, 1758 is found all over the Indo-West Pacific and commonly associated with coral reef habitats and diet algae (Fleure and Gettings 1907; Purcell and Ceccarelli 2021). Top shells (*Trochus* species) hold significant economic value, not only as a traditional food source but also as an important export commodity, with their aragonite shells widely used in the manufacture of mother-of-pearl buttons and ornamental products (Gillett et al. 2020).

Since the 1750s, Linnaeus laid the foundation for species classification by describing numerous taxa. Subsequently, Dillwyn applied this classification system to the genus *Trochus*, thereby advancing the taxonomic research of this group (Dillwyn 1817). Although many studies have provided additional images and morphological descriptions of *Trochus* species, the absence of clear morphological illustrations has left the issue of accurate species identification unresolved (Philippi 1845; Reeve 1860; Dodge 1958). Taxonomic research on *Trochus* in China began in the 1930s, when Zhang Xi conducted morphological and ecological studies on *Trochus maculatus* (Zhang 1962). The Taiwanese malacologist Lai Jingyang also contributed to the taxonomic understanding of the genus *Trochus* (Lai 1981). Based on the earlier studies, and Dong (2002) described nine *Trochus* species and identified a new species, *T. zhangi*. In recent years, studies focusing on the morphology of the genus *Trochus* have been scarce. Notably, Sawayama et al. (2022) carried out a detailed investigation of *T. histrio* and *T. rota* from Japan, analyzing both morphological traits and molecular markers to assess variation within and between species.

The genus *Trochus* is highly diverse, and species-level identification is often challenging (Cunha et al. 2023). Some *Trochus* species, such as *T. firmus* and *T. erythreus*, or *T. flammulatus* and *T. maculatus*, exhibit highly similar shell morphologies, making it difficult to distinguish them based solely on external features (Tryon et al. 1898). Traditionally, species identification and description have relied primarily on morphological characteristics. With the development of molecular and imaging techniques, species delimitation has become more efficient and accurate. Among these, DNA barcoding plays a key role in identifying newly discovered species (Stoeckle 2003). However, an increasing number of taxonomic studies have shown that some species exhibit highly intraspecific shell variation and lack clear original descriptions, making image-based records alone insufficient for accurate taxonomic identification (Affenzeller et al. 2017). The emergence and development of 3D modelling techniques have enabled the extraction of key morphological traits by providing more comprehensive and detailed shape information. Moreover, 3D morphological data are becoming increasingly cost-effective and accessible for species-level studies, including intra-specific variation. For example, previous research has combined DNA barcoding and 3D morphological analyses to delimit species within the genus *Pyrenaearia* (Caro et al. 2019).

In this study, we diagnose and describe two new *Trochus* species from the South China Sea. We apply multiple species delimitation methods to evaluate genetic divergence among *Trochus* species based on mitochondrial cytochrome *c* oxidase subunit I (COI) sequences. Additionally, we update the 3D models and extract detailed morphological characteristics of the new *Trochus* species, providing a valuable reference for future comparative taxonomic studies.

## ﻿Material and methods

### ﻿Sample collection

**Figure 1.**
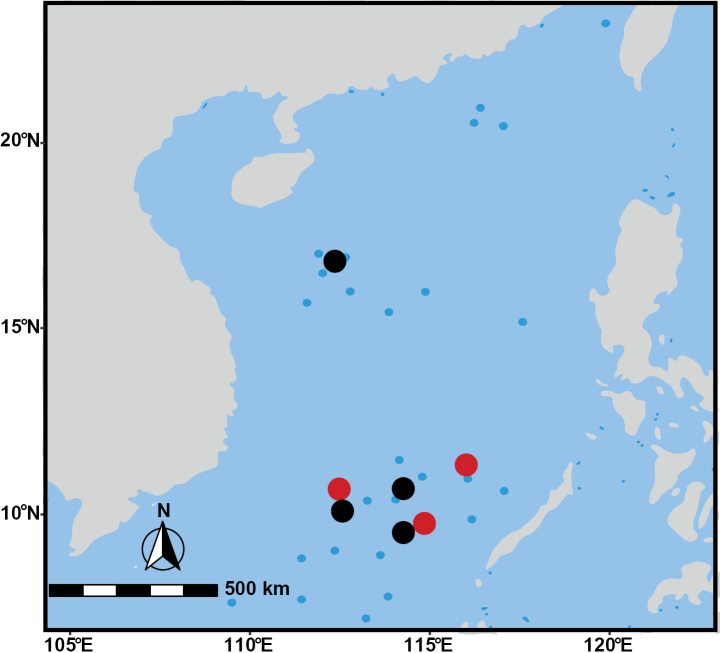
Sampling locations of *Trochus* species in the South China Sea. Red dots indicate collection sites of *T. parvus* sp. nov., while black dots indicate sites of *T. nanhai* sp. nov. The blue dots indicate the locations of islands.

Specimens were collected from coral reef habitats in the South China Sea (Fig. 1). Adductor muscle tissues from freshly collected individuals were flash-frozen in liquid nitrogen and stored at -80 °C for subsequent DNA extraction. The remaining parts of the specimens were preserved at -20 °C and deposited in the Laboratory of Intertidal Ecophysiology, Qingdao, China.

### ﻿Morphological observation and 3D model construction

Specimens were soaked in 5% hydrogen peroxide for 5 minutes to remove organic residues. The remaining crustose coralline algae were then carefully scraped off using a spatula. All specimens were photographed using a Nikon D3500 camera equipped with an AF-S macro lens (Nikon, Tokyo, Japan). Image stacks of standard views of the shells (Callomon 2019) were produced and combined using Helcon Focus v. 7.7.0. All photographs were enhanced in Adobe Photoshop 2019 (Adobe Systems, San Jose, CA, USA). The 3D models were constructed using Agisoft Metashape. To ensure comprehensive coverage, each specimen was rotated and photographed from multiple angles. For each specimen, photographs were aligned to determine camera positions and generate a sparse point cloud. Subsequently, depth maps were generated from the sparse point cloud and produced the final 3D model. From these models, we extracted shell morphometric parameters, including Ellipticity, Normalized Avg Curvature, and Sphericity, using the workflow described by Yan et al. (2024). All feature extraction procedures were based on Python scripts available on GitHub (https://github.com/yanchengrui123/Feature-extraction). Shell height and width were measured manually using traditional caliper measurements in the field. The radulae were collected following the method described by Han et al. (2024) and examined using a Scanning Electron Microscope (SEM) after being thinly coated with gold.

### ﻿DNA Extraction and PCR Amplification

DNA was extracted from muscle tissue using the CTAB protocol (Ding et al. 2018), and the COI gene was amplified with the universal primers LCO1490 and HCO2198 (Folmer et al. 1994). PCR reactions were performed in 25 µL volume, each containing 1 μL DNA and 24 uL PCR mix. The PCR mix comprised 12.5 μL of 2× Taq PCR Mix (Tiangen, Beijing), 1 μL of each 10 μM primer, and 9.5 μL of DNase-free ddH_2_O. PCR was performed with the following cycling conditions: 95 °C for 2 min; 30 cycles of 95 °C for 50 s, 45 °C for 50 s, and 72 °C for 50 s; followed by a final extension at 72 °C for 5 min. PCR amplicons were submitted to Sangon Biotech (Shanghai, China) for bidirectional sequencing using the same primer pair. We manually checked the quality of the sequence peak map and used SeqMan (DNASTAR Inc., USA) to assemble the forward and reverse sequences.

### ﻿Phylogenetic analyses

The 24 newly generated COI sequences in this study were subjected to BLAST searches against the GenBank database to assist in grouping and taxonomic assignment of the specimens. To clarify the systematic status of *Trochus* specimens in this study, 30 *Trochus*COI sequences were retrieved from GenBank (see Suppl. material 1: table S1) to construct the phylogenetic trees. *Coelotrochus viridis* (Gmelin, 1791) GQ249683, *Clanculus margaritarius* (Philippi, 1846) PP652117, and *Tectarius cumingii* (Philippi, 1846) AJ488640 were selected as outgroup taxa. Alignment of COI sequences was conducted with MAFFT v. 7 (Katoh and Standley 2013) and ModelFinder was employed to identify the optimal substitution model (Kalyaanamoorthy et al. 2017). Maximum likelihood (ML) analysis was conducted using IQ-TREE with the GTR+F+I+G4 model and 1000 bootstrap replicates (Nguyen et al. 2015). Bayesian inference (BI) was performed using MrBayes with the GTR+I+G+F model, run for 50,000,000 generations with a burn-in of 25% (Ronquist et al. 2012). The resulting phylogenetic trees were visualized using iTOL (Letunic and Bork 2024).

Multiple species delimitation methods were used to determine whether the newly identified species is genetically distinct from other congeners. Aligned COI sequences were submitted to the Automatic Partitioning (ASAP) website (https://bioinfo.mnhn.fr/abi/public/asap/asapold.html) using the Kimura (K80) model with a transition/transversion (ts/tv) ratio of 2.0 (Puillandre et al. 2021). The ASAP analysis provides partitioning scores, and the partition with the lowest score was selected as the optimal species delimitation. ABGD analysis was conducted using aligned sequences as input on the online platform (https://bioinfo.mnhn.fr/abi/public/abgd/abgdold.html; Puillandre et al. 2012), with the K2P model selected and a relative gap width set to 1.0. The ML phylogenetic tree was used as input for species delimitation analysis using the Bayesian implementation of the Poisson Tree Processes (bPTP) method on the online server (https://species.h-its.org/ptp/), with default parameters. Species delimitation using the General Mixed Yule-Coalescent (GMYC) model (Pons et al. 2006) was performed in R with the splits package (Ezard et al. 2009), based on ultrametric trees generated in BEAST2 under the Yule model. The maximum number of putative species inferred from delimitation analyses was used to define species groups, and pairwise *p*-distance comparisons among groups were calculated using MEGA 11 (Kumar et al. 2018).

## ﻿Results

### ﻿Molecular analysis

**Figure 2.**
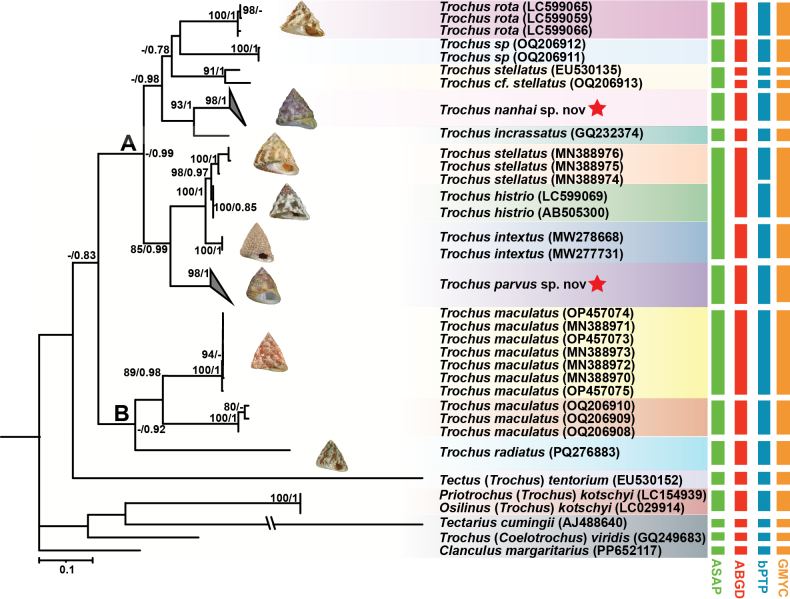
Maximum likelihood (ML) and Bayesian inference (BI) phylogenetic trees of *Trochus* based on COI sequences. Bootstrap support values above 70% (ML) and posterior probabilities above 0.7 (BI) are shown. The color bar on the right indicates species clusters inferred by four species delimitation methods.

COI sequences (679 bp) of the newly sequenced *Trochus* specimens do not match any known *Trochus* species (GenBank: *T. nanhai* sp. nov. (PX057741–61) *T. parvus* sp. nov. (PX058089–91). The BI and ML trees shared the same topology and comprised two major clades. However, the BI tree showed higher support values, particularly along the main branches (Fig. 2). The two new *Trochus* species were significantly different from other *Trochus* genetic lineages and were placed on two distinct branches within clade A. These results were further supported by genetic distance and morphological characteristics (see Remarks under the new species description).

Species delimitation results varied among methods. ASAP identified 15 species, both ABGD and GMYC recovered 17 species, while bPTP delimited 18 species. The greatest variation in species delimitation occurs within clade A. All species delimitation methods consistently placed *Trochus maculatus* within clade B and supported its genetic divergence from other closely related species with high confidence. In the ASAP analysis, the sequence originally labeled as *T. stellatus* (MN388974–76) in GenBank was resolved within the clade containing *T. histrio* and *T. intextus*. Additionally, *T. stellatus* (OQ206912) and *T. cf. stellatus* (OQ206913) were identified as the same species by ASAP. However, ABGD, bPTP, and GMYC analyses all supported the separation of these two sequences as distinct species. Both ABGD and GMYC identified *T. stellatus* and *T. histrio* as a single species, whereas bPTP distinguished them as two separate species. All four species delimitation methods identified the *Trochus* species and the newly proposed species as distinct molecular operational taxonomic units (MOTUs) or putative species.

**Figure 3.**
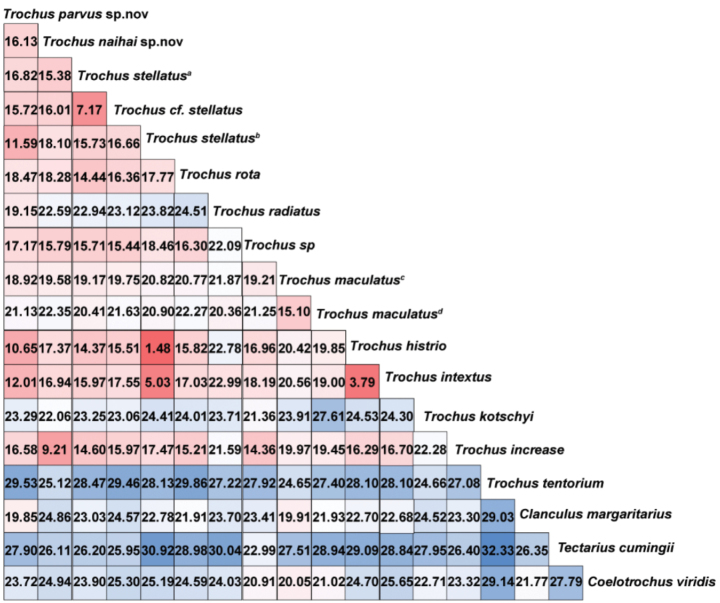
Mean Kimura 2-parameter (K2P) pairwise genetic distances (%) among groups identified by the bPTP species delimitation method. Note: a: EU530135; b: MN388974–76; c: MN388970–73; d: OQ206908–10.

*Trochus parvus* sp. nov. and *T. nanhai* sp. nov. exhibited interspecific K2P distances ranging from 10.65% to 29.53% and 9.21% to 25.12%, respectively, when compared with other congeners. While the mean K2P genetic distances within the newly described species were 0.23% for *T. parvus* sp. nov. and 0.72% for *T. nanhai* sp. nov. The observed interspecific K2P distances were more than ten times greater than the intraspecific distances, consistent with the commonly applied “10× rule” for species delimitation. *Trochus stellatus*^a^ showed relatively low mean K2P distances of 1.48% and 5.03% with *T. histrio* and *T. intextus*, respectively. *Trochus histrio* exhibited 3.79% K2P values with *T. intextus* (Fig. 3). The average intraspecific K2P distance of *Trochus* species based on COI sequences ranged from 0% to 0.72%, indicating low and relatively stable genetic divergence within species (Table 1).

**Table 1.** The average intraspecific Kimura 2-parameter (K2P) distance (%) was calculated based on COI sequences of *Trochus* species.

Species	Distance	S.E.
*Trochus histrio*	0	0
*Trochus rota*	0.54	0.27
*Trochus stellatus* ^a^	0.42	0.21
*Trochus parvus* sp. nov.	0.23	0.1
*Trochus maculatus* ^b^	0.13	0
*Trochus maculatus* ^c^	0.1	0.1
*Trochus* sp.	0.15	0.16
*Trochus nanhai* sp. nov.	0.72	0.26
*Trochus intextus*	0	0
*Trochus kotschyi*	0	0

## ﻿Systematics

### ﻿Order Trochida

**Superfamily Trochoidea Rafinesque, 1815**

**Family Trochidae Rafinesque, 1815**

#### ﻿Genus Trochus

Taxon classificationAnimaliaGastropodaTrochidae

Linnaeus, 1758

F08A4E96-5C42-5139-B69E-05E0542903D1

##### Type species.

*Trochus maculatus* Linnaeus, 1758; Recent, Indo-Pacific region.

#### ﻿ Trochus nanhai sp. nov.

Taxon classificationAnimaliaGastropodaTrochidae

178CD295-891C-50CA-99B0-484F44A39B6F

https://zoobank.org/52CD3716-4C4C-4514-8B08-EECB758B2B0C

##### Etymology.

The name “nanhai” is derived from the Chinese designation for the South China Sea, the only region where this species has been documented to date.

##### Material examined.

***Holotype***: LINE-SCSLH-20240601005, Complete specimen, deposited in the Laboratory of Intertidal Ecophysiology, Ocean University of China (OUC), Qingdao, China (Suppl. material 1). ***Paratype***: LINE-SCSZQ-20240531001, LINE-SCSYSZ-20240521002, LINE-SCSMJ-20240501004 and LINE-SCSAL-20240601001, Complete specimen, same location as holotype (Suppl. material 1).

##### Description.

**Figure 4.**
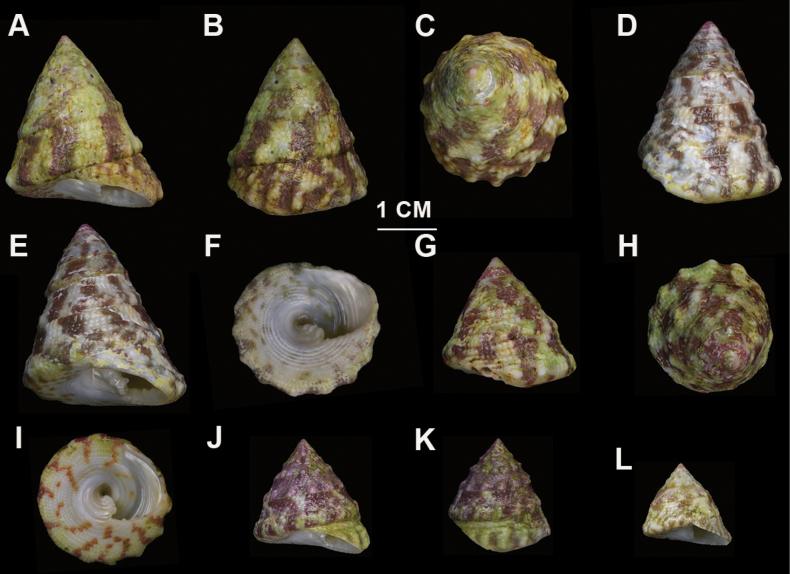
*Trochus nanhai* sp. nov. A–C. Specimen LINE-SCSZQ-20240531001, elate-conic. A. Shell in apertural view; B. Shell in lateral view; C. Shell in apical view; D–F. Elate-conic specimen LINE-SCSYSZ-20240521002; D. Shell in lateral view; E. Shell in apertural view; F. Shell in ventral view; G–I. Specimen LINE-SCSLH-20240601005, low-conic; G. Shell in lateral view; H. Shell in apical view; I. Shell in ventral view. J, K. Specimen LINE-SCSMJ-20240501004, low-conic. J. Shell in apertural view; K. Shell in lateral view; L. Shell in apertural view, specimen LINE-SCSAL-20240601001, small subadult low-conic.

***Shell***: medium size, elate-conic and low-conic, solid and heavy, and falsely umbilicate. Spire composed of 7–8 planulate whorls, with 4–5 regular closely spaced spiral rows of uniform granules on each whorl (Fig. 4A–K). These granules are rounded, bead-like, or slightly compressed. Each whorl periphery bears prominent, evenly spaced pustules; these may be inconspicuous in small subadults (Fig. 4L). In elate-conic specimens, the whorl pustules gradually fuse during growth, resulting in smoother and more continuous beaded spiral ridges (Fig. 4A–E). The periphery of the body whorl exhibits 12–15 distinct longitudinal folds forming oblong nodules. The base of the shell is a little concave, sculptured with 11–12 concentric granulose lirae (Fig. 4F, I). The upper surface bears distinct, broad reddish-brown longitudinal stripes, approximately equal in width to the alternating whitish bands. The base bears a narrow row of spots corresponding to the outer colour pattern. The inner lip is thickened with 3–4 folds. The columella bears 4–5 plicate dentates (Fig. 4A, E, F, I, J). Internally, the shell displays a pearly luster accompanied by distinct spiral lirae.

***Radula*. **Radula rhipidoglossate, radular formula n × 5 × 1 × 5 × n (Fig. 6A). The central tooth with U-shaped cusp, broad triangular shaft, symmetrically flanked by eight denticles (Fig. 6B). Five lateral teeth are present on each side, each with a fold on the shaft that tightly interlocks with the shaft of the adjacent tooth, and bears 3–5 denticles on one side only. The fifth lateral tooth bears a distinct paddle-shaped cusp. Marginal teeth are narrow and sickle-shaped; the innermost 10–11 marginal teeth each bear 2–3 denticles (Fig. 6C). From the 11^th^ to 12^th^ marginal teeth onwards, tooth size gradually decreases, while the number of denticles increases, forming comb or feather-like structures (Fig. 6D).

##### Type locality.

Neritic zone of the South China Sea; Coral reef.

##### Measurements.

Shell height 12.9–27.8 mm, shell width 14.0–24.9 mm, ellipticity 1.56E+08–4.45E+10, Normalized Avg Curvature 0.055–0.46, sphericity 0.69–0.79, (*N* = 17). (Details are shown in Suppl. material 1: table S3).

##### Remarks.

The genus *Trochus* is widely distributed across coral reef habitats throughout the Indo-West Pacific. Based on its conical shell, falsely umbilicate and radula morphology, *T. nanhai* sp. nov. is assigned to the genus *Trochus*. Due to its coral reef habitat, the shell surface is often encrusted with crustose coralline algae, which obscures key morphological features and makes species identification more difficult. Upon laboratory examination, the elate-conic specimens, characterized by beaded spiral ridges, were found to resemble *T. calcaratus*. Although the morphological descriptions and illustrations are outdated, the species can still be distinguished based on the original descriptions, illustrations, and figures provided by Tryon et al. (1898) and Herbert (1996). *Trochus nanhai* sp. nov. differs from *T. calcaratus* in having fewer whorls (6–7 vs. 9), a base bearing densely granose lirae (11–12 vs. 6–7), and fewer peripheral pustules (12–15 vs. up to 28 on the body whorl). Comparisons with available image resources further reveal that *T. nanhai* sp. nov. possesses fewer but more pronounced and sharply defined pustules along the whorl periphery (Suppl. material 1: fig. S1A–C). Similar characters, particularly the number and prominence of knob-like structures on the shell base, have also been used as reliable diagnostic features for distinguishing *Trochus* species (Sawayama et al. 2022). Notably, *T. nanhai* sp. nov. lacks the fistulous or perforated peripheral tubercles and spiniform tubulose structures that are present in *T. calcaratus*. The subadult shell of the new species resembles that of the type species, *T. maculatus*. However, in *T. maculatus*, bears 6–8 spiral beaded liræ on each whorl, in contrast to the 4 regular spiral rows of uniform granules on each whorl observed in the new species. The radula of *Trochus* species has also been poorly described in previous studies (Fasila and Vinod 2024). In the present study, however, the marginal teeth of *T. nanhai* sp. nov. are more clearly defined: the first 10–11 marginal teeth are sickle-shaped, each bearing 2–3 denticles. Detailed morphological differences between *T. nanhai* sp. nov., *T. sacellum*, *T. calcaratus*, and the phylogenetically related *T. stellatus* are provided in Suppl. material 1: table S2.

#### ﻿ Trochus parvus sp. nov.

Taxon classificationAnimaliaGastropodaTrochidae

03F4DC9C-AE6E-5DA5-AB52-C58DAE6E6757

https://zoobank.org/95BCDB2D-FEB3-41D7-9736-DFE358687E48

##### Etymology.

The specific epithet “*parvus*” is derived from the Latin, meaning “small”, and refers to the relatively diminutive shell size of the new species, which is a remarkable difference from other species in this genus.

##### Material examined.

***Holotype***: LINE-SCSZQ-20240531002, Complete specimen, deposited in the Laboratory of Intertidal Ecophysiology, Ocean University of China (OUC), Qingdao, China (Suppl. material 1). ***Paratype***: LINE-SCSMJ-20240501007, Complete specimen, same location as holotype (Suppl. material 1).

##### Description.

**Figure 5.**
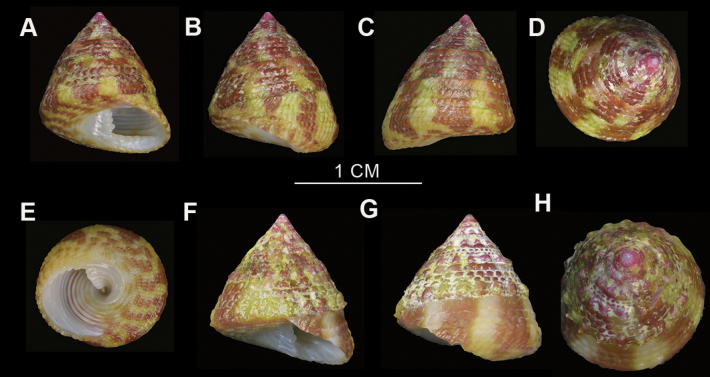
*Trochus parvus* sp. nov. A–D. Specimen LINE-SCSZQ-20240531002. A. Shell in apertural view; B. Shell in lateral view; C. Shell in dorsal view; D. Shell in apical view. E–H. Specimen LINE-SCSMJ-20240501007. E. Shell in ventral view; F. Shell in apertural view; G. Shell in lateral view; H. Apical view of shell.

***Shell***: Small in size, conical, with an acute apex. The shell surface bears prominent reddish-brown longitudinal stripes that taper into spots toward the base; the apex is typically red. Composed of 6 planulate whorls, with 3–4 spiral rows of closely arranged granules (Fig. 5A–D, F–H). These granules are rounded, bead-like, or slightly compressed, and the upper whorls are smaller than those on the lower whorls. The periphery of the whorls bears a spiral ridge composed of prominent, evenly spaced pustules. The body whorl is sculptured with compact, nearly quadrate, flattened beads, forming a rounded profile. The base is slightly concave, with 7–8 concentric granulose lirae, separated by interstices as wide as the ridges (Fig. 5E). The columella is oblique, bearing 3–4 closely packed plicate-dentate, forming a false umbilicus. The inner lip contains 4 folds (Fig. 5A, E, F). The inner shell surface is nacreous with fine spiral lirae.

**Figure 6.**
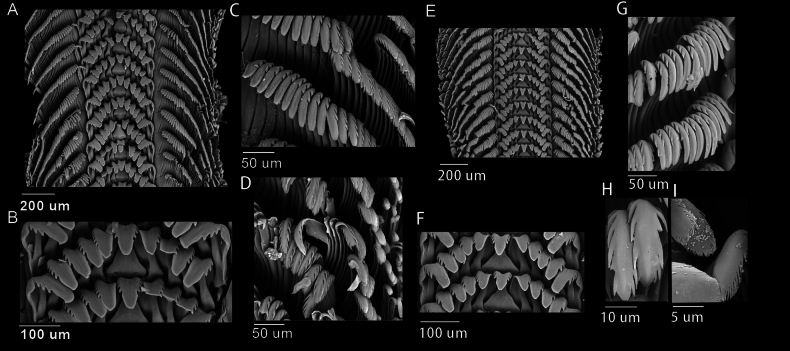
Radula of *Trochus nanhai* sp. nov. and *T. parvus* sp. nov. A–D. *T. nanhai* sp. nov. A. Entire radular row; B. Rachidian tooth; C. Innermost marginal tooth; D. Outermost marginal tooth. E–I. *T. parvus* sp. nov. E. Entire radular row; F. Rachidian tooth; G. Innermost marginal tooth; H. Comb-like marginal structures; I. Feather-like marginal structures.

***Radula*. **Radula rhipidoglossate, radular formula n × 5 × 1 × 5 × n (Fig. 6E). Central tooth with V-shaped cusp, broad trapezoid shaft, symmetrically flanked by eight denticles (Fig. 6F). Five lateral teeth are present on each side, each with a fold on the shaft that tightly interlocks with the shaft of the adjacent tooth, bears 3–5 denticles on one side only. The fifth lateral tooth bears a distinct paddle-shaped cusp. Marginal teeth are narrow and sickle-shaped; innermost 9–12 marginal teeth each bear 2–3 denticles (Fig. 6G). From the 10^th^ to 13^th^ marginal teeth onwards, tooth size gradually decreases, while the number of denticles increases, forming a comb (Fig. 6H) or feather-like structures (Fig. 6I).

##### Type locality.

Currently, the Neritic zone of the South China Sea; Coral reef.

##### Measurements.

Shell height 11.0–13.0 mm, shell width 10.4–14.0 mm, ellipticity 1.77E+08–1.88E+08, Normalized Avg Curvature 0.087–0.19, sphericity 0.72–0.80, (*N* = 2). Details in Suppl. material 1: table S3.

##### Remarks.

Morphological characteristics support the validity of *T. parvus* sp. nov. as a species distinct from the type species *T. maculatus* (Fig. 5; Suppl. material 1: fig. S1D, E). The shells of *T. parvus* sp. nov. are relatively small compared with other *Trochus* species. Like *T. nanhai* sp. nov., *T. parvus* sp. nov. exhibits a conical shell, falsely umbilicate and radula morphology, which are in accord with the key characteristics of *Trochus*. However, the small size of *T. parvus* sp. nov. may indicate a subadult stage, possibly due to a shorter lifespan, and its shell shows minimal encrustation by crustose coralline algae. In *T. maculatus*, the shell consists of 7–8 whorls, each whorl bearing 6–8 spiral beaded lirae, and the body whorl distinctly angulated periphery. The base of the shell is radiately marked with discontinuous black lines forming a maculated or finely tessellated pattern, and is concentrically sculptured with about 10 fine, slightly beaded lirae (Suppl. material 1: fig. S1D, E). In contrast, *T. parvus* sp. nov. has fewer whorls (6 vs. 7–8) and the base of the shell is concentrically sculptured (7–8 vs. 10), has a rounded body whorl (rounded vs. carinated), and the base bears a radiating series of reddish-brown dots forming irregular, interrupted lines.

Molecular data further support the distinctiveness of *T. parvus* sp. nov. from *T. maculatus*. The two species form well-supported, separate clades (A and B), and the mean K2P genetic distance between them reaches 18%, thereby confirming their separation at the species level (Figs 2, 3). Despite being genetically close to *T. histrio*, *T. parvus* sp. nov. is clearly separable by its carinated body whorl and other morphological differences (Sawayama et al. 2022).

## ﻿Discussion

We identified two new *Trochus* species based on an integrative approach combining morphological characters and molecular analyses. These species exhibit distinct morphometric features and group into well-supported clades in the phylogenetic trees. Additionally, the interspecific K2P genetic distances based on COI sequences exceed intraspecific variations, consistent with the widely accepted “10× rule” for species delimitation (Hebert et al. 2003). As a cutting-edge tool for morphological analysis, 3D modeling has proven useful for studying both interspecific differences and intraspecific variation (Sánchez González et al. 2025). To better characterize morphological features and quantify intraspecific variation, we constructed 3D models of the two newly described species. The extracted morphological parameters will serve as a valuable reference for future taxonomic and evolutionary studies of *Trochus*.

The radula plays an important role in taxonomic studies and often reveals intraspecific differences more effectively than shell morphology (Mutaf and Aksit 2009). The radular formula of the new species is the same as that of other *Trochus* species (Ashja Ardalan et al. 2023; Fasila and Vinod 2024). In this study, radular morphology is largely similar between the two new *Trochus* species, with only slight differences observed in the shape of the central tooth cusp, the number of denticles on the fifth lateral tooth, and the position at which the marginal teeth begin to decrease in size and increase in denticle number. Similar patterns have been observed in other Trochidae species. In those studies, despite considerable genetic divergence among species, the radular structure remains largely conserved (Yamamori and Kato 2018; Zhang et al. 2020).

Multiple species delimitation methods (ASAP, ABGD, bPTP, and GMYC) were used to identify specimens and test species hypotheses, consistently recognizing two new *Trochus* species as distinct. However, discrepancies among methods suggest the presence of cryptic species or potential misidentifications of the genus *Trochus*. The ASAP method provides the most conservative results among species delimitation and identifies the fewest molecular operational taxonomic units (MOTUs) (Heimburger et al. 2022). In contrast, the same K2P model distance-based method, ABGD identified 17 MOTUs. Monophyly-based methods such as bPTP and GMYC use DNA sequence data directly to infer species boundaries, identifying 18 and 17 species, respectively. Previous studies suggest that monophyly-based methods may avoid the loss of genetic information that can occur when sequences are converted to pairwise distances and are therefore considered to be more effective in certain cases (Kerr et al. 2009). These findings suggest the possible presence of cryptic species and the limitation of current taxonomy. Molecular species delimitation methods have been shown to sometimes misinterpret intraspecific population structure as interspecific divergence, potentially leading to an overestimation of species diversity (Lohse 2009; Sukumaran and Knowles 2017). In our study, this issue is exemplified by *T. stellatus*, *T. histrio*, and *T. intextus*, which occupy three well-supported, distinct positions in the phylogenetic tree, despite exhibiting relatively low K2P genetic distances (1.48–5.03%). As shown in Suppl. material 1, *T. stellatus* was collected from Hainan, while *T. histrio* originated from Japan. Due to insufficient morphological descriptions and a lack of images, possible taxonomic misidentifications in the original submissions cannot be confirmed. Nonetheless, our results suggest that some of these previous records may indeed represent misidentifications.

*Trochus nanhai* sp. nov. exhibits two shell morphologies, elate-conic and low-conic. The most prominent feature of this species is the presence of peripheral pustules along the whorl edges. These pustules are especially prominent in low-conic individuals (where shell height is less than shell width, SH < WH). In contrast, in elate-conic individuals, the pustules tend to gradually fuse, forming smoother and more strongly beaded spiral ridges. This pattern is consistent with previous findings in *T. histrio*, where intraspecific variation in shell morphology correlated with SH. The basal knobs of *T. histrio* tend to become smoother as the shell grows (Sawayama et al. 2022).

The South China Sea, as a biodiversity hotspot, harbors many previously unrecorded species. In this study, we identified and described two new *Trochus* species based on both morphological and molecular data. Meanwhile, we detected misidentifications in previous studies. To help prevent such errors in the future, we constructed 3D models of the new species. We propose that 3D morphological comparisons should be adopted as an important reference in future *Trochus* taxonomy.

## Supplementary Material

XML Treatment for Trochus

XML Treatment for Trochus nanhai

XML Treatment for Trochus parvus

## ﻿Additional information

### Use of AI

No use of AI was reported.

### Author ORCIDs

Peng-Jin Zhu https://orcid.org/0009-0001-3126-4899

Li-Sha Hu https://orcid.org/0000-0003-4669-8654

Yun-Wei Dong https://orcid.org/0000-0003-4550-2322

### Data availability

All of the data that support the findings of this study are available in the main text or Supplementary Information.

## Supplementary materials

Supplementary material 1 Supplementary tables and figure Data type docx https://binary.pensoft.net/file/1496426This dataset is made available under the Open Database License (http://opendatacommons.org/licenses/odbl/1.0/). The Open Database License (ODbL) is a license agreement intended to allow users to freely share, modify, and use this Dataset while maintaining this same freedom for others, provided that the original source and author(s) are credited.Peng-Jin Zhu, Cheng-Rui Yan, Hong-Qiang Yang, Li-Sha Hu, Yun-Wei Dong
