# Supplementary material for: ﻿Description of two new species of the genus Trochus Linnaeus, 1758 (Gastropoda, Trochidae) from the South China Sea
Source: Zookeys. 2025 Dec 19;1264:265–80. doi: 10.3897/zookeys.1264.167854 (PMC12743251; doi:10.3897/zookeys.1264.167854)
Supplement: Supplementary material 1 — Supplementary tables and figure [file zookeys-1264-265_article-167854__-s001.docx]

**Supplementary material**

Table S1. Voucher information, GenBank accession numbers, and collection localities for all sequenced *Trochus* specimens.

| Species | Specimen  Voucher | Locality | Latitude / Longitude | GenBank/BOLD  Accession |
| --- | --- | --- | --- | --- |
| *Trochus parvus* sp. nov | LINE-SCSMJ-20240501007 | Meiji, Sansha,  Hainan, China | 115.44^∘^E,  9.57^∘^N | PX058090 |
| *Trochus parvus* sp. nov | LINE-SCSYS-20240515001 | Yongshu, Sansha,  Hainan, China | 112.96^∘^E,  9.61^∘^N | PX058091 |
| *Trochus parvus* sp. nov | LINE-SCSZQ-20240531002 | Zhuquan, Sansha,  Hainan, China | 114.59^∘^E,  9.98^∘^N | PX058089 |
| *Trochus nanhai* sp. nov | LINE-SCSAD-20240605001 | Anda, Sansha,  Hainan, China | 114.70°E, 10.35°N | PX057759 |
| *Trochus nanhai* sp. nov | LINE-SCSZQ-20240531001 | Zhuquan, Sansha,  Hainan, China | 114.59^∘^E,  9.98^∘^N | PX057750 |
| *Trochus nanhai* sp. nov | LINE-SCSYSZ-20240521001-2 | Yongshu, Sansha,  Hainan, China | 112.96^∘^E,  9.61^∘^N | PX057749/51 |
| *Trochus nanhai* sp. nov | LINE-SCSYS-20240515002 | Yongshu, Sansha,  Hainan, China | 112.96^∘^E,  9.61^∘^N | PX057748 |
| *Trochus nanhai* sp. nov | LINE-SCSXM-20240522001-2 | Ximen, Sansha,  Hainan, China | 114.39°E, 9.90°N | PX057741-2 |
| *Trochus nanhai* sp. nov | LINE-SCSMJ-20240501001-6 | Meiji, Sansha,  Hainan, China | 115.44^∘^E,  9.57^∘^N | PX057752-57 |
| *Trochus nanhai* sp. nov | LINE-SCSMJ-20240501008 | Meiji, Sansha,  Hainan, China | 115.44^∘^E,  9.57^∘^N | PX057758 |
| *Trochus nanhai* sp. nov | LINE-SCSBK-20240604001 | Baikou, Sansha,  Hainan, China | 114.52°E,  9.87°N | PX057761 |
| *Trochus nanhai* sp. nov | LINE-SCSAL-20240601001 | Anle, Sansha,  Hainan, China | 114.52°E,  9.93°N | PX057760 |
| *Trochus nanhai* sp. nov | LINE-SCSLH-20240601001-4,6 | Langhua, Sansha, Hainan, China | 112.55°E,  16.05°N | PX057743-47 |
| *Trochus rota* | - | Nagai, Kanagawa Prefecture, Japan | 139.60°E,  35.20°N | LC599059/65/66 |
| *Trochus* sp. | - | Malé, Kaafu Atoll, Maldives | - | OQ206911/12 |
| *Trochus cf. stellatus* | - | Malé, Kaafu Atoll, Maldives | - | OQ206913 |
| *Trochus stellatus* | - | Benoki, Okinawa Pref., Japan | - | EU530135 |
| *Trochus incrassatus* | - | South of Yate, southeastern New Caledonia, French | - | GQ232374 |
| *Trochus stellatus* | - | Hainan island,  Hainan, China | - | MN388974-76 |
| *Trochus histrio* | - | Nagai, Kanagawa Prefecture, Japan | 139.60°E,  35.20°N | LC599069 |
| *Trochus histrio* | - | Marukihama, Bounotsu, Kagoshima Pref.Japan | - | AB505300 |
| *Trochus intextus* | - | - | - | MW278668/31 |
| *Trochus maculatus* | - | Hainan island,  Hainan, China | - | OP457073-5 |
| *Trochus maculatus* | - | - |  | MN388970-72 |
| *Trochus maculatus* | - | Malé, Kaafu Atoll, Maldives |  | OQ206908-10 |
| *Trochus radiatus* | - | - | - | PQ276883 |
| *Tectus* (*Trochus*) *tentorium* | - | Coconut Beach, Lizard Island, Queensland, Australia | - | EU530152 |
| *Priotrochus* (*Trochus*) *kotschyi* | - | - | - | LC154939 |
| *Osilinus* (*Trochus*) *kotschyi* | - | - | - | LC029914 |

Table S2. Morphological comparison among eight *Trochus* species with close genetic relationships or similar shell morphology.

| Species | Shell-size | Sculpture on shell | Whorls | Inner lip and columella |
| --- | --- | --- | --- | --- |
| *Trochus parvus* sp. nov | Small-sized | The whorls bear 3-4 rows of regularly arranged small beads. The periphery of the whorl features a spiral ridge composed of prominent, evenly spaced pustules. The body whorl is sculptured with closely packed, relatively flattened, subquadrate beads. Base shell bearing 7-8 concentric, densely granose lirae. | 6 whorls; planulate; body whorl rounded; suture pustules. | 3-4 plicate-dentate; The columella is oblique and plicate, irregularly 4 folds. |
| *Trochus nanhai* sp. nov | Medium-sized | The whorls bear 4-5 rows of regularly arranged small granules. The periphery of each whorl is ornamented with prominent pustules. The body whorl exhibits 12-15 longitudinal folds forming oblong nodules. Base shell bearing 11-12 concentric, densely granose lirae. | 7-8 whorls; planulate; body whorl carinated; suture pustules. | 3-4 plicate-dentates; The columella is oblique and plicate, irregularly 4 folds. |
| *Trochus stellatus*（*increase*） | Medium-sized | The whorls bear 5-6 spiral rows of granules, and the peripheral margin of each whorl is ornamented with radiating spinose projections. Base shell bearing 7 concentric, densely granose lirae. | 7-8 whorls; whorls are slightly convex; body whorl rounded; suture spinous. | 4-5 plicate-dentates; The columella is oblique and plicate, irregularly 4-5 folds. |
| *Trochus histrio* | Medium-sized | The whorl is sculptured with 3 to 4 rows of bead-like granules. The peripheral margin bears hollow spinose projections, some of which terminate in rounded tips. | 7-8 whorls; slightly convex; body whorl carinated; suture spinous. | 3-4 plicate-dentates; The columella is oblique and plicate, irregularly 3-4 folds. |
| *Trochus maculatus* | Medium-sized | The shell surface bears 6 to 8 spiral bead-bearing lirae. The beads are sometimes shaped like longitudinal folds, and sometimes appear as distinct, rounded granules. The base of the shell is concentrically sculptured with about 10 fine, variably beaded lirae. | 7-8 whorls; planulate or concave; body whorl exhibits a pronounced angulated periphery; suture impressed. | 4-5 plicate-dentates; The columella is plicate, irregularly 4-5 folds. |
| *Trochus sacellum* | Medium-sized | Shell with six spiral cinguli: 1st prominent with paired confluent tubercles; 2nd-3rd with discrete tubercles; 4th–5th with radiating costae; 6th alternating spiniform tubercles and beads. Last whorl peripherally carinated with 18 crenulated nodes. Base shell bearing 8 concentric, densely granose lirae. | 8 whorls; planulate or concave; body whorl carinated; suture impressed. | 3-4 plicate-dentates; The columella is oblique and plicate, irregularly 3-4 folds. |
| *Trochus calcaratus* | Medium-sized | Whorls with 4-5 spiral rows of granules; periphery with radially aligned pustules bearing minute perforations; body whorl sharply carinated with 28 pustules. Base shell bearing 6-7 concentric, densely granose lirae. | 9 whorls; planulate; body whorl carinated; suture pustules. | 4 plicate-dentates; The columella is oblique and plicate, irregularly 4 folds. |
| *Trochus intextus* | Medium-sized | 5-6 rows of rounded granules per whorl; smaller on body whorl. | 8 whorls; slightly convex; body whorl obtusely angulated; suture concave | 4-5 plicate-dentates; The columella is oblique and plicate, irregularly 4 folds. |

Table S3. 3D Measurements of *Trochus parvus* sp. nov. and *Trochus nanhai* sp. nov..

| **Specimen**  **Voucher** | Height (mm) | Width (mm) | Ellipticity | Normalized Avg Curvature | Sphericity |
| --- | --- | --- | --- | --- | --- |
| LINE-SCSZQ-20240531002 | 11.3 | 10.4 | 1.77E+08 | 0.087 | 0.80 |
| LINE-SCSMJ-20240501007 | 13.0 | 14.0 | - | - | - |
| LINE-SCSYS-20240515001 | 11.0 | 13.8 | 1.88E+08 | 0.19 | 0.72 |
| LINE-SCSZQ-20240531001 | 27.3 | 24.9 | 4.45E+10 | 0.38 | 0.69 |
| LINE-SCSYSZ-20240521001 | 23.0 | 24.0 | 1.82E+08 | 0.09 | 0.72 |
| LINE-SCSYSZ-20240521002 | 27.8 | 24.7 | 3.49E+10 | 0.31 | 0.70 |
| LINE-SCSMJ-20240501004 | 20.1 | 22.9 | 6.29E+09 | 0.22 | 0.71 |
| LINE-SCSAL-20240601001 | 13.5 | 14.0 | 3.42E+10 | 0.46 | 0.71 |
| LINE-SCSAD-20240605001 | 22.9 | 20.8 | 1.61E+08 | 0.13 | 0.78 |
| LINE-SCSXM-20240522001 | 20.5 | 24.6 | 1.56E+08 | 0.07 | 0.75 |
| LINE-SCSXM-20240522002 | 21.6 | 23.5 | 1.95E+08 | 0.08 | 0.79 |
| LINE-SCSMJ-20240501001 | 23.0 | 22.2 | 1.59E+10 | 0.36 | 0.72 |
| LINE-SCSMJ-20240501002 | 19.0 | 19.9 | 1.67E+08 | 0.11 | 0.72 |
| LINE-SCSMJ-20240501006 | 12.9 | 16.6 | 1.71E+08 | 0.13 | 0.75 |
| LINE-SCSLH-20240601001 | 19.0 | 23.9 | 1.83E+08 | 0.22 | 0.74 |
| LINE-SCSLH-20240601002 | 22.0 | 24.1 | 1.81E+08 | 0.24 | 0.74 |
| LINE-SCSLH-20240601003 | 22.7 | 24.0 | 1.61E+08 | 0.09 | 0.78 |
| LINE-SCSLH-20240601006 | 17.6 | 19.6 | 1.57E+08 | 0.05 | 0.75 |
| LINE-SCSLH-20240601007 | 20.1 | 24.4 | 1.78E+08 | 0.07 | 0.75 |
| LINE-SCSBK-20240604001 | 19.7 | 20.1 | 1.69E+08 | 0.09 | 0.72 |


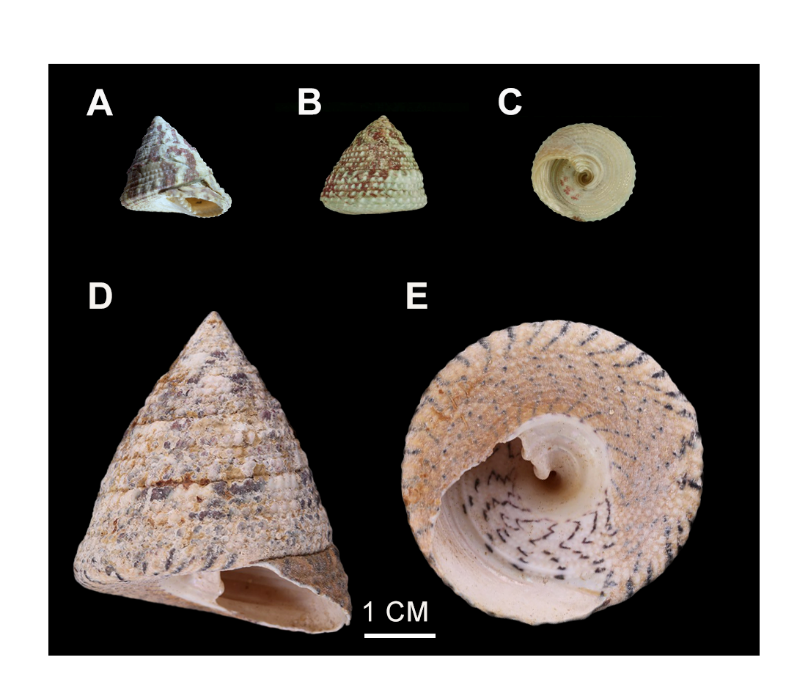


Fig. S1. Available image resources of *Trochus calcaratus* Souverbie, 1875 and *Trochus maculatus* Linnaeus, 1758. A, apertural view of *T. calcaratus* (Kyoto University Museum, specimen no. 75). B-C, apertural and ventral view of *T. calcaratus* (Taiwan Malacofauna Database). D-E, apertural and ventral view of *T. maculatus* (National Animal Collection Resource Center, specimen no. SCSMBC002890).
